# Annotations

**Published:** 1889-03-09

**Authors:** 


### ANNOTATIONS.

**March Dust.**

"A PECK of March dust is worth a king's ransom." How many of our learned professors and "crabbed" examiners could tell us the why and wherefore of this old agricultural proverb? Our ancestors knew some things well, and we know a good many of which they knew nothing. But in learning the new things we have permitted many of the old to be forgotten. We are becoming a people of books, not of things. Our forefathers knew things better than books. Why is "March dust" so priceless? Because it is the proof and evidence of a clear, dry atmosphere. And why is a clear, dry atmosphere so essential in March? Because the soil can be ploughed and harrowed, and the seed put into the ground in a dry, clean condition. Seed thus sown gives the best promise of a thriving and fruitful crop. Dry winds in March are priceless for the soil. Moreover, in our northerly counties dry, clear weather is helpful for the young lambs, which at this season are beginning to put in an appearance. The cold does them little harm when the ground and the atmosphere remain dry. March weather, if not wet, is favourable both to vegetable and animal life, so long as that life is carried on under sound conditions. But how stands the question when we turn our attention to man? Rustic man shares the health of the lambs and the crops in a dry March. Urban man, the miserable town-dweller, droops and shivers, coughs and wheezes, groans over his torpid liver, and bans March as the most execrable month of the year. If health and comfort are to be made possible for the town man, he must either change himself or change the British climate. The question whether of the two will have to be changed is not so difficult as to demand a reference to the Royal Society. Even the brilliant Tyndal or the dogmatic Huxley would hesitate before they attempted to change what the latter once called our "pagan climate." March, it is certain, will not be converted to better ways. Then the town-dwelling man must convert himself if he wants to be well. What is the modus operandi? Physic? Emphatically no! If the enemy is to be conquered he must be faced. Shivering indoors is the basest and most un-English of surrenders. Flannels and walking-sticks are the weapons of war. He who clothes himself properly and walks six miles a-day all the year round may bid defiance to the "bleak winds of March."

**The Horse and his Murderers.**

OUR country cousins last week assembled in their tens of thousands at the Agricultural Hall, to see and admire and consider how they might still further improve that noble animal the horse. Of four-footed creatures the horse, for beauty, strength, speed, and serviceableness, is *facile princeps*. His intelligence is second only to that of the dog, if, indeed, it be not equal to it. The people who value and make much of so noble and docile a creature, by that very fact show themselves possessed of a certain nobility of mind. In this country the horse is held to be the most royal of animals, and our countrymen have just reason to be proud of the fact. Honouring the horse they honour themselves. But whilst the horse is the friend and admired companion of the countryman, what is the position he is reduced to in the town? There, even under the most favourable circumstances, he is generally a complete stranger to his master. It is true the latter will occasionally mount him for a ride of "show" or "ceremony," and will sometimes condescend to sit behind him in a victoria, or a lumbering brougham, or landau, whilst the gorgeous coachman spreads himself out on the box, and holds the reins with all the solemnity of a sexton holding the rope with which he is letting down a coffin. That, however, is a mere trifle, although it means almost the complete annihilation of the fine intellect of the animal. What do the servants do who have practically the sole control of their masters' horses? What, above all, do the vestries and other public bodies do, who make and repair the roads on which the horse has to do his work? How can a man speak with any calmness of the shameful and imbecile cruelties to which horses of all kinds are subjected in frosty or rainy weather? Here is a coal waggon, with two tons of coal inside, and one horse yoked to it, hopelessly striving to draw the load up Highgate Hill, or other steep road, whilst the ground is as slippery as a waxed floor. No sooner is the City reached than the face reddens with shame as the "bus" and "dray" horses flounder and sprawl in the vain effort to make their way onward, and every now and then fall helplessly on the hard ground with a thud which makes the heart sick. Of course we must have roads, and smooth roads, especially for the City; but is it beyond the utmost power of the "resources of civilisation" to make the roads of all London rough and safe on a frosty morning, or the City streets clean and non-slippery on a rainy and greasy day? The thing is ludicrous. Your countryman, who is held to be less clever than his town brother, is not such a fool as to unnerve and slowly murder his valuable horse in that mad way. If he cannot get his roads "roughed," he gets his horse "roughed," and thus the claims of humanity and of self-interest are both satisfied. Every driver of a horse should himself be able to "rough" the animal under his care, and should be made to do so. Or, if that be not possible, every owner should be compelled to have his horses "roughed" in slippery weather before they leave the stable-yard. But until that is done, the vestries and others who are responsible for the care of the roads should feel that they are no better than horse murderers so long as they leave the roads and streets in their present intolerable condition.

**"Horseflesh" to the Fore.**

THE philosopher who reflects upon the length of time it has taken to induce a small portion of the public to believe that the eating of horseflesh is not really a horrifying act, not indeed half so horrifying as eating one's grandmother would be, can well understand why it should have taken uncounted millions of ages to develop from a little portion of Huxley's friend *Bathybius* the body of a W. G. Grace or the mind of a Herbert Spencer. But Herbert Spencer's mind and W. G. Grace's body are here at last, and so is the practice of eating horseflesh. We have more than once expressed a decided opinion in these pages on the wholesomeness of good, sound horseflesh and on its fitness for human food, and therefore we need not now repeat that opinion. It is indeed not a question which the opinion of any person can materially affect. No man of sense waits upon opinion if he has the means at hand of making an experiment. Horseflesh has been used by many people for years as food, and is found to be both palatable and nuritious. Scientific men did not of course need to wait for the result of an experiment. The "place of the horse in Nature" was quite sufficient to substantiate the fact of his wholesomeness as food. The actual sale of horses, asses, and mules by what we must call "butchers" has expanded to very considerable dimensions; so much so as to require strict regulation. A Bill has been printed, and is to be brought before the House of Commons at the first convenient opportunity. The provisions are of a very simple but also of a very necessary kind. It is taken for granted that horseflesh will be sold, but it is only to be sold as horseflesh. Every seller of it is to have his shop or place of business adequately labelled, with letters not less than four inches long, as a place where horseflesh is sold. He is not to sell the article unless asked for it, except on the condition that each portion bears a printed label indicating its true description. Medical officers of health are to have full powers of inspection and seizure. For the purposes of the Act, asses and mules are to be raised to the dignity of horses. Perhaps, as their new honours will be of a purely posthumous character, the "asses and mules," if consulted, would prefer to remain in the humbler sphere they now occupy, and so to save at once their scientific autonomy and their lives. The subject is one of no very paramount interest for the public except as helping to demonstrate that the doctrine of development has really a basis in fact. Our grandfathers, and especially our grandmothers, would not have eaten horseflesh. It would have made the "gorge" rise too unpleasantly. We know better. Science has come to our aid, and has bidden this one infinitesimal prejudice vanish, and it is slowly vanishing. For so much comfort our gratitude is necessarily considerable. Perhaps in due time we shall rise to the intellectual eminence of the South Sea Islanders, and without qualm of any kind, make a comfortable meal off a dead friend or even off a fine plump ancestor! Who shall say?

